# Retroviruses of the Human Virobiota: The Recycling of Viral Genes and the Resulting Advantages for Human Hosts During Evolution

**DOI:** 10.3389/fmicb.2020.01140

**Published:** 2020-05-28

**Authors:** Anna Luganini, Giorgio Gribaudo

**Affiliations:** Laboratory of Microbiology and Virology, Department of Life Sciences and Systems Biology, University of Turin, Turin, Italy

**Keywords:** human endogenous retrovirus, symbiotic relationship, evolution, syncytin, beneficial functions

## Abstract

All humans are colonized by a vast diversity of microbes (bacteria, archaea, protozoa, yeast, and fungi; collectively referred to as the microbiota) and viruses (the virobiota). This latter group includes viruses infecting prokaryotic cells (bacteriophages), viruses infecting eukaryotic-host cells, and virus-derived genetic elements present in host chromosomes. Although these eukaryotic viruses are mostly known to be pathogens, they are also able to establish mutualistic relationships with humans. Little is known about the mutualistic aspects of viral infection. Nevertheless, it is clear that evolution of some animal virus-host interactions has led to benefits in the health of the hosts, as is the case with symbiogenesis and endogenization of retroviruses that has exerted a neuroprotective effect on the human brain, and an important role in the fetal development, thus on the evolution of host species. In this review, we summarize how retroviruses provide amazing examples of cooperative-evolution, i.e., successful exchange between viruses and host, and how, in some cases, the benefits have become essential for the hosts’ survival.

## Introduction

**FIGURE 1:**
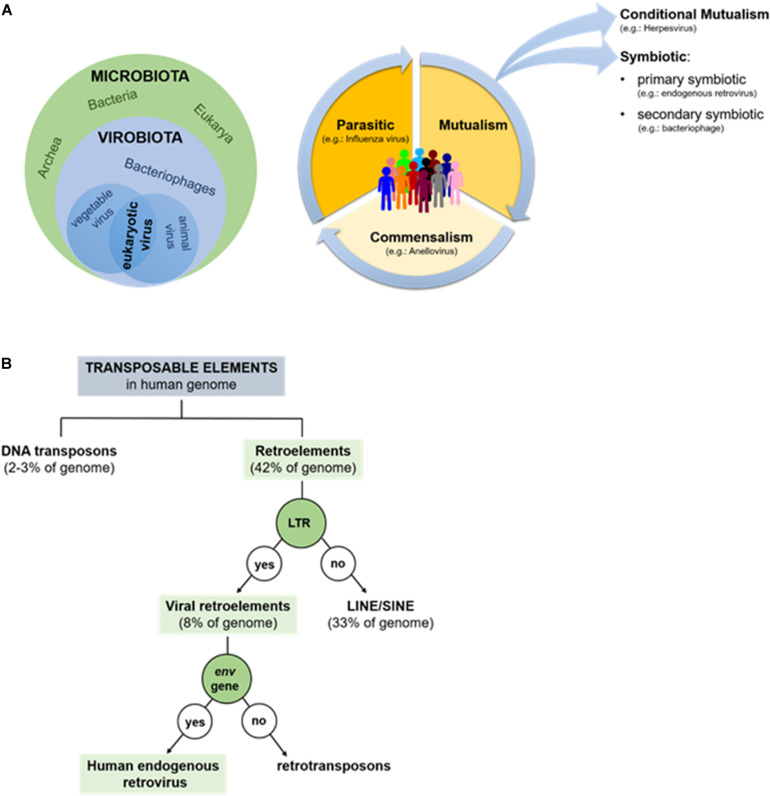
**(A)** Human microbiota and virobiota. The green circle contains the components of the microbiota (Bacteria, Archea, and Eukaria), while the blue-light contains all the members of the virobiota (bacteriophages, eukaryotic viruses), which in turn is included in the microbiota (on left). Types of dynamic interaction between microorganisms and human-host: the community can shift from parasitism to mutualism and commensalism, favoring an improved state of health; on the contrary if microorganisms shift toward parasitism, they favor the disease (on right). **(B)** Repetitive mobile sequences present in human genome: almost half of genome is made up by transposable elements: DNA transposons (2–3%) and retroelements (42%). The former amplifies without RNA, the latter requires a reverse transcriptase.

Recent metagenomics studies have revealed that, not only is the human body colonized by microorganisms belonging to all three biological domains (i.e., Eukarya, Archaea, and Bacteria), but that the human microbiota is also composed of many viruses (Handley et al., 2012; Duerkop and Hooper, 2013). As such, the term “microbiota” also comprises the term “virobiota,” a huge community of viruses that science has only just begun to explore and whose physiological roles are still unknown (White et al., 2012; Figure 1A). The viruses present in the human virobiota are bacteriophages (infecting bacteria) and eukaryotic viruses, which replicate in animal cells or that infect plants associated with the host’s diet. Thanks to the presence of this last category of viruses the virobiota is considered a dynamic community that varies based on what each of us eats and where we live (Freer et al., 2018). While we might consider some bacteriophages as stable residents of the human body, since they infect the bacteria that stably colonize the human host (Allen and Abedon, 2013), the situation is quite different with regard to eukaryotic viruses. In this latter case, metagenomic studies have found it very difficult to discriminate truly resident viruses associated with the host from viruses whose presence is due to acute (e.g., influenza virus), chronic (e.g., hepatitis B virus), or latent infections (e.g., herpesvirus) (Duerkop and Hooper, 2013).

According to the canonical definition, viruses are parasites associated with negative consequences; in other words, pathogenic viruses cause diseases in the human host, who either survives or dies as a consequence (e.g., influenza virus, rabies virus, and measles virus). Only recently, has it been shown that among the 220 viruses able to infect humans, only about 100 are pathogenic (Parker, 2016). The remainder is composed of apathogenic viruses that establish commensal or symbiotic interactions with host (Figure 1A). To the best of our knowledge, anelloviruses (AV) could provide the prototypical example of a human commensal virus and are also a major component of the virobiota. This virus family includes a group of single stranded DNA-viruses, newly discovered (in 1997), that are present in both the blood and tissues of most humans, where they replicate persistently without causing disease (Freer et al., 2018).

A key example of a symbiotic virus-host relationship, on the other hand, is provided by the human endogenous retroviruses (HERVs). A symbiotic interaction is a form of mutualism whereby both virus and the human host gain benefits from their relationship (Figure 1A). More specifically, HERVs are primary symbionts because the viruses, after infecting germ cells, are transmitted vertically from parent to child (Parker, 2016). Although some evidence exists indicating that HERVs could contribute to the insurgence of certain pathologies [e.g., multiple sclerosis (MS), carcinogenesis, or bipolar disorder; Antony et al., 2004; Galli et al., 2005], in this review, we will focus on the beneficial effects of HERVs on humans. HERVs in fact provide an excellent example of how a “negative” event, such as a viral infection, can have a positive impact on the host’s biology, establishing a successful interaction with it.

## Human Endogenous Retroviruses (Hervs)

Data provided by the Human Genome Project suggest that the most abundant repetitive class of human DNA sequences is made up of distinct families of transposable elements (about 46–47% of human genome), of which 2–3% correspond to DNA transposons and 42–43% to retroelements (Smit, 1999). Based on the presence or absence of a long terminal repeat (LTR), retroelements are then divided into two large groups: non-LTR retroelements (corresponding to 33% of the human genome) and including LINEs (long interspersed nuclear elements) and SINEs (short interspersed nuclear elements) (Larsson and Andersson, 1998), and LTR-containing viral retroelements (around 8% of the human genome) that consist of HERV and retrotransposons, which differ from each other by the presence/absence of *env* gene (Figure 1B; Nelson et al., 2004).

Human endogenous retroviruses represent therefore a group of transposable elements that were originally acquired through the exogenous infection of primate hosts by ancestor retroviruses over the course of primate evolution (Katzourakis et al., 2009; Hayward et al., 2015), i.e., between 5 and 70 million years ago (Aiewsakun and Katzourakis, 2015). In contrast with exogenous retroviruses that are horizontally transmitted between hosts and usually associated with disease, HERVs infections have affected both somatic cells and germ-lines. Hence, following an occasional process of endogenization and further fixation, through which viral DNA copies permanently integrated into the host’s germ-line chromosomes, HERVs were vertically transmitted to progeny in a Mendelian fashion, determining their coevolution with the host genome (Katzourakis et al., 2009; Lee et al., 2013). As such, once endogenized, HERVs retain their similarities with the ancestral exogenous virus for an exceedingly long time (millions of years) since they incur point mutations at the same rate of the host, about 10^–9^ substitutions per site per year (s/n/y) instead of a virus’s rate of 10^–3^ s/n/y (Hanada et al., 2004; Sanjuán, 2012). Ancestral HERVs integrated into the human genome around 35–45 million years ago, during the split between Old and New World monkeys (Bannert and Kurth, 2006). Only HERV-K viruses are the most recently integrated group (occurring ~5 million years ago); they are exclusive to humans and contain the most complete proviral sequences.

**FIGURE 2:**
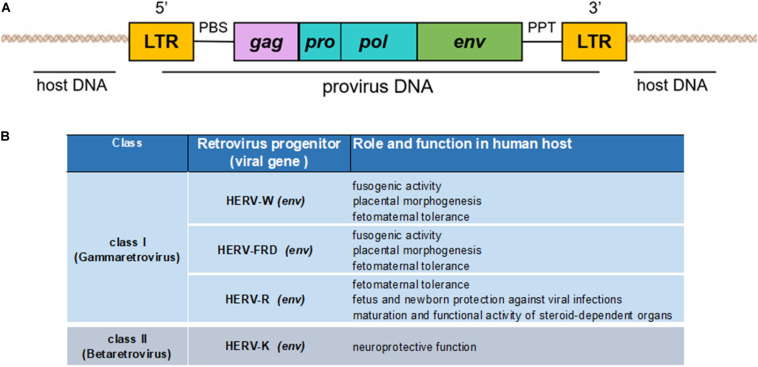
**(A)** Genomic organization of an endogenous retrovirus. The integrated double-stranded DNA is characterized by the long terminal repeats (LTRs) at its 5′ and 3′ ends. LTRs flank the retroviral *gag, pro/pol*, and *env* genes. **(B)** Schematization of the beneficial effects of HERVs to human host.

Human endogenous retroviruses have a genomic organization similar to that of the exogenous retroviruses, composed of the viral genes *gag*, *pro-pol*, and *env*, flanked by two LTRs (Figure 2A). LTRs are formed during the reverse transcription of the viral RNA genome and regulate the viral genes’ expression due to the presence of promoters, enhancers, and polyadenylation signals (Schön et al., 2009; Vargiu et al., 2016). The *gag* gene encodes three structural virion components: the matrix (MA), capsid (CA), and nucleocapsid (NC) proteins. The *pol* gene encodes two enzymes necessary for the viral life cycle: reverse transcriptase/integrase (*pol*) and protease (*pro*), which synthesize the complementary DNA from the viral RNA and enable proviral integration. The *env* gene encodes two envelope proteins: transmembrane protein (TM) and surface protein (SU) (Larsson and Andersson, 1998), which mediate virus entry into and egress from host cells.

Human endogenous retroviruses can sometimes harbor more than these four retroviral genes, like type I HERV-K, whose *env* gene gives rise to two splice variants: the accessory proteins Np9 and Rec (Armbruester et al., 2002; Grandi et al., 2017), or type II HERV-K with Sp and Rec (Ruggieri et al., 2009; Douville and Avindra, 2014). Between the 5′LTR and *gag*, a primer-binding site (PBS) is located, and a polypurine trait (PPT) lies between *env* and the 3′LTR. Both these sequences localized between the LTRs exert important functions: the cellular tRNA becomes bound to the PBS during the reverse transcription process, whereas the PPT is the primer for (+) strand DNA production. Moreover, the PBS sequence has traditionally been used for a systematic nomenclature of HERV, since HERV group names are generally identified using a letter that characterizes the human tRNA type that binds to the viral PBS sequences during the retrotranscription process of the viral genome (e.g., HERV-K for lysine, HERV-W for tryptophan, etc.). If the PBS sequence is not available, HERV groups are also named according to unconventional criteria, such as the name of a neighboring gene (HERV-ADP), a clone number (e.g., HERV-S71), or a particular amino acid motif (HERV-FRD) (Werner et al., 1990; Lyn et al., 1993; Seifarth et al., 1995). However, this characterization of the HERV groups at the genomic level is considered inadequate and incomplete because univocal guidelines for the naming of groups are missing (Grandi and Tramontano, 2018b). Recently, a new multi-step approach based on a phylogenetic study (considering the highly conserved *pol* gene) and univocal taxonomic rules allowed the splitting up of about 3200 HERV insertions, identified using the RetroTector program (ReTe) (Sperber et al., 2007), into 39 “canonical” groups plus other 31 “non-canonical” clades (Vargiu et al., 2016). Nonetheless, conventional classification also divides each HERV group into three classes according to sequence homology with exogenous retroviruses: class I (Gammaretrovirus- and Epsilonretrovirus-like: HERV-E, -F, -H, -I, -P, -R, -T, -W, and HERV-FRD, etc.); class II (Betaretrovirus-like: HERV-K); and class III (Spumaretrovirus-like: HERV-L) (Larsson and Andersson, 1998).

Hosts infected by HERVs can adopt various strategies to repress the viral replication and prevent the horizontal transmission of infections between individuals, such as viral genome silencing through epigenetic regulation mechanisms (DNA methylation) or the association of histones to viral DNA to limit its accessibility to the proteins involved in gene transcription (Grandi and Tramontano, 2018b). When viral DNA is integrated into the human host genome, it can undergo over time recombination events leading to the formation of a solitary LTR, or to insertions, deletions, and mutations, thus inactivating the ability of HERVs to produce infectious viruses. In this regard, one of the more ancient components of the cellular machinery that confer intrinsic immunity against mobile elements is represented by the APOBEC cytidine deaminases. Among these cellular enzymes, members of the APOBEC3 (A3) protein family induce G-to-A hypermutations in HERV genomes, thus leading to suppression of HERV replication and its mobility in favor of the host’s genomic integrity (Ito et al., 2020).

However, as we will see, this is not always the case, because several HERVs have been co-opted into physiological functions of the host, with some members being actively transcribed and differentially expressed depending on host cell type and the physiological circumstances (Figure 2B).

## Herv and the Placenta

In some cases, retroviral genes are recycled since they confer an advantage to the host: the most famous examples are syncytin-1 and syncytin-2. Syncytin-1 and -2 are functional glycoproteins that mediate cell-cell fusion between cytotrophoblasts and placental syncytiotrophoblasts localized at the fetomaternal interface, which plays a fundamental role in the exchange of nutrients, hormones, and gases between the mother and the fetus and is required for normal embryonic growth (Muyan and Boime, 1997; Handwerger and Freemark, 2000). Additionally, syncytiotrophoblasts keep the maternal immunosuppressive state under control, preventing fetal rejection (Munoz-Suano et al., 2001; Nakamura, 2009). It has been known for some years that the syncytin-1 sequence is homologous to that of the *env* gene of the complete HERV-W provirus (Perron et al., 2005); whereas syncytin-2 corresponds with the HERV-FRD-*env* gene.

It is probable that the syncytin proteins of retroviruses enable membrane fusion with host cells, facilitating viral infection. This function has been “commandeered” in placental mammals as it makes the placenta more invasive toward the maternal uterus, thus conveying an evolutionary advantage to the embryo. Similarly to other retroviral glycoproteins, syncytin-1 and syncytin-2 are synthesized as precursors that only become functional after their cleavage into two functional subunits: SU (involved in cellular receptor binding) and TM (involved in cell-cell fusion). Both proteins also harbor an immunosuppressive domain (ISD) that, as mentioned above, is required for generating the fetomaternal tolerance state during pregnancy, although the molecular mechanisms involved in the modulation of the immune response are not known (Lokossou et al., 2014).

High expression of HERV-R (class I retrovirus) also helps protect the fetus from the maternal immune response. In fact, its expression results in elevated concentrations of env protein that is potentially immunosuppressive. A direct correlation has been identified between the expression levels of fusogenic syncytin-1 and -2 and preclampsia (or gestosis) events, since both proteins levels were shown to be decreased in preclampsia following hypermethylation in the 3′LTR (Vargas et al., 2011). Considering this strong correlation, these HERV proteins could be used as potential markers for the early diagnosis of preclampsia.

In addition to this known function in placentation in mammals, a recent study has shown that syncytin also supports myogenesis, increasing muscle mass in male mice (Redelsperger et al., 2016). In fact, deletion of the syncytin B isoform led to a 20% reduction in muscle mass in knockout mice compared with animals with both syncytin A and syncytin B. This reduction was male-specific because not visible in female. As suggested by the authors of the study, syncytin B could play a decisive role in the muscular development of male mice, and its role may explain the muscle sexual dimorphism observed in all placental mammals (Redelsperger et al., 2016).

## Herv and Brain Protection

A number of HERVs (e.g., -W, -K, -R, and -E) are expressed in human brain tissues (Antony et al., 2006, 2007), but a neuroprotective function was only ascribed to class II HERV-K *env* gene. In fact, deep transcriptomic sequencing analyses revealed significantly greater HERV-K expression in healthy fetal brain than all other HERVs (Bhat et al., 2014). Using an *in vitro* paradigm, the same study showed that transfection of the human neuronal cell line (SK-N-SH) with an HERV-K*env* expression plasmid enhanced the transcription of the neurotrophins nerve growth factor (NGF) and brain-derived neurotrophic factor (BDNF) compared with cells transfected with a control construct (pGFP). Both these neurotrophins contribute toward the growth and survival of developing neurons and to the maintenance of neuronal function (Bhat et al., 2014). Indeed, it has been proposed that their depletion might lead to the development of CNS disorders (Allen et al., 2013). Bhat and colleagues also showed HERV-K *env* to confer neurotrophic effects on neuronal cells exposed to neurotoxins, as observed in murine NG108 neuronal cells transfected with pHERV-K*env* and treated with different neurotoxins, staurosporine and HIV-1 Vpr protein, in which the cellular viability reached 100 and 60%, respectively, in response to the treatment. These results substantiate a beneficial effect of HERV-K on neuronal cells by preventing neuronal injury mediated by these two neurotoxins (Bhat et al., 2014). A neuroprotective effect of HERV-K was also demonstrated *in vivo* by means of a neurobehavioral study performed with immunodeficient HIV-vpr/RAG1^–^/^–^ mice able to express the neurotoxic Vpr protein. For this experiment, neuronal stem cells (NSCs) were transfected with pHERV-K *env* or pGFP, as control, and then implanted into the striatum of the animals. In line with the results of the *in vitro* experiment, NSCs cells expressing HERV-K env protein showed high levels of BDNF without any changes in neuronal morphology or density. Moreover, diminished tumor necrosis factor alpha (TNFα) expression (a well-known toxic factor) was observed together with reduced neuroinflammation. Striatal function was also evaluated by assessing the rotational behavior of the animals caused by a lesion resulting from the unilateral intrastriatal injection of amphetamine (Bhat et al., 2014). To summarize briefly, following the induced neurological injury, mice would exhibit ipsiversive movement toward the side of the lesion; in terms of their rotary behavior, the mice implanted with pHERV-K *env*-transfected cells exhibited reduced neurobehavioral deficits respect to mice implanted with the control construct. The results of this *in vivo* experiment strengthen the *in vitro* data obtained so far on the neuroprotective properties exerted by HERV-K *env*.

## Herv and Hormone-Dependent Organs

Another possible role of HERV *env*-encoded proteins could be their involvement in the maturation and functional activity of steroid-dependent organs. As in the case of the placenta, HERV-R expression is particularly high during fetal development and it is thought to be engaged in embryogenesis (Andersson et al., 2002). A previous study conducted on three developing fetuses (5–6 weeks) demonstrated an organ-specific expression of HERV-R, since high levels of HERV-R *env* mRNA in the secretary epithelium of the gut, the kidneys, the tongue, the heart, the liver, and the central nervous system, but not in the epidermidis were observed. However, the highest level of expression was demonstrated in the placenta and in the adrenal cortex (Andersson et al., 2002). In the latter case, env protein was also detected using immunofluorescence in a postpartum fetus. This expression may be controlled by the presence of androgens that regulate HERV-R expression, since this retrovirus contains androgen receptor (AR) sites in its 5′LTR (Kato et al., 1987). In fact, it is known that about half way through pregnancy the adrenal cortex secretes androgens necessary for the growth and development of the fetus (O’Rahilly, 1983), which, could, in turn, bind to the retroviral AR sites. Moreover, since high levels of HERV-R have also been reported in human adult adrenal cortex (Katsumata et al., 1998), we might suppose that *env* expression is constitutive and not limited to embryogenesis period. Although a role remains to be established in human adult, the most accredited hypothesis is that HERV-R could be involved in protecting the fetus and newborn against further retroviral infections through the mechanism of receptor interference. In fact, since the sebum makes up part of the vernix caseosa that covers the fetus’s skin, it is possible that the presence of HERV-R env protein in this secretion is able to prevent a viral infection by blocking the corresponding receptor (Andersson et al., 1996). This hypothesis may be reasonable for female subjects, but a potential function of HERV-R *env* expression in steroid hormone-dependent male organs remains to be discovered.

## Herv and Antiviral Activity

During viral infections, host cells activate different restriction factors, coping with such infections. In the context of exogenous retrovirus infections, more than nine groups of cellular restriction factors have been reported to interfere with Human Immunodeficiency Virus type 1 (HIV-1) replication. They include the well-characterized APOBEC3G, SAMHD1, Tetherin/BST-2, and TRIM5α proteins (Johnson, 2013), and those more recently characterized such as MX-2, SERINC3/5, IFITMs, Schlafen 11, and MARCH2/8 (Goujon et al., 2013; Zhang et al., 2018). Relevant to this, endogenized HERVs can be potentially considered *per se* as restriction factors able to exert protective effects against exogenous retroviruses (Grandi and Tramontano, 2018a). Three possible mechanisms have been suggested by which HERV could promote resistance to exogenous retrovirus infections: (1) Occurrence of complementary interactions between HERV mRNAs and homologous RNAs originated by exogenous retrovirus, with formation of dsRNA molecules that, in turn, can stimulate the Toll-Like Receptor 3 (TLR3) and thus an innate immune response (Zhou et al., 2010). (2) Aggregation of HERV and retroviral proteins, as observed in cells co-infected by both HIV-1 and HERV-K, in which gag proteins of both viruses colocalized at the plasma membrane and co-assembly into the same HIV-1 virions, thus inhibiting release of new HIV-1 infectious particles (Monde et al., 2017). (3) Superinfection interference, as that exerted by HERV pseudo-viral particles or HERV-derived proteins that block retrovirus entry through cellular-receptor interference. This was the case of the truncated HERV-F env protein (lacking the TM subunit) encoded by the *suppressyn* gene, that by binding the cell receptor ASCT2 could prevent the entry of several type D-retroviruses (Sugimoto et al., 2013).

Eventually, a negative effect of the full-length HERV-K 108 env protein was observed *in vitro* on the release of infectious HIV-1 virions production, albeit further studies are needed to elucidate the underlying molecular mechanism, it seems that it does not belong to any of the above described (Terry et al., 2017).

However, despite the above *in vitro* observations, there is not yet direct evidence that the 30 *env* genes found in the human genome confer resistance to retroviruses *in vivo* (Johnson, 2013).

## Conclusion

Human endogenous retroviruses are an integral part of the human genome and result from ancestral infections by their exogenous progenitors of cells of the human germline, where they have undergone replication together with host genes. Theoretically, the presence of these viral sequences could be potentially harmful for the host because their integration could lead to mutations. However, HERVs were not eliminated during evolution, but rather they were maintained in the host-genome. If, in the past, HERVs were mistakenly considered as useless elements of the human genome (DNA junk), today some are recognized as conferring biological advantages. In fact, in some cases, HERV genomes have undergone a process of positive selection during evolution, being exploited by the hosts to benefit important physiological processes.

As we have summarized in this review, HERVs appear to play important roles in physiology, fetal development, and human evolution: if the accidental infection of a mammalian ancestor by an exogenous retrovirus had never occurred, the placenta and the mammals that produce it, including humans, would never have existed. These beneficial consequences can explain why HERVs have been “fixed” into the genome instead of being eliminated over the years. Nevertheless, the endogenization of retroviruses was not without consequences: during evolution, the majority of HERVs accumulated deletions and mutations that generally compromised their ability to replicate and produce proteins unless under specific conditions. This was the price to be paid for their survival. In this review, we have reported on current findings regarding the biological advantages that HERVs confer to humans. Indeed, endogenous retroviruses provide a perfect example of a symbiotic relationship between a virus and its host, yet a profound understanding of HERV biology that could lead to a better comprehension of their roles is still missing. However, some HERVs are also compatible with parasitic relationship. In fact, various studies have highlighted an association between the presence of HERVs, probably those that have integrated into human genome more recently, and some rare and incurable diseases, in particular autoimmune, neurodegenerative, and cancer diseases (Antony et al., 2004; Grandi and Tramontano, 2017, 2018a; Ibba et al., 2018). These findings are highly relevant because, if a clear evidence emerges that HERVs are explicitly involved in the onset of these pathologies, this would open up new treatment prospects, as they would no-longer be considered incurable diseases, but as pathologies involving one or more retroviruses against which antiretroviral therapies could be used or developed. Indeed, this knowledge would pave the way to important new therapeutic approaches for the treatment of such pathologies.

## Author Contributions

AL conceptualized and wrote the manuscript. GG revised the manuscript. All authors gave final approval of the version to be submitted and any revised version.

## Conflict of Interest

The authors declare that the research was conducted in the absence of any commercial or financial relationships that could be construed as a potential conflict of interest.
